# Bruton’s tyrosine kinase inhibition attenuates disease progression by reducing renal immune cell invasion in mice with hemolytic-uremic syndrome

**DOI:** 10.3389/fimmu.2023.1105181

**Published:** 2023-02-23

**Authors:** Sarah Kröller, Bianka Wissuwa, Sophie Dennhardt, Nadine Krieg, Christoph Thiemermann, Christoph Daniel, Kerstin Amann, Florian Gunzer, Sina M. Coldewey

**Affiliations:** ^1^ Department of Anesthesiology and Intensive Care Medicine, Jena University Hospital, Jena, Germany; ^2^ Septomics Research Center, Jena University Hospital, Jena, Germany; ^3^ William Harvey Research Institute, Barts and The London School of Medicine and Dentistry, Queen Mary University of London, London, United Kingdom; ^4^ Department of Nephropathology, Friedrich-Alexander University (FAU) Erlangen-Nürnberg, Erlangen, Germany; ^5^ Department of Hospital Infection Control, University Hospital Carl Gustav Carus, TU Dresden, Dresden, Germany; ^6^ Center for Sepsis Control and Care (CSCC), Jena University Hospital, Jena, Germany

**Keywords:** hemolytic-uremic syndrome, acute kidney injury, inflammation, animal model, Bruton’s tyrosine kinase, ibrutinib, acalabrutinib

## Abstract

Hemolytic-uremic syndrome (HUS) can occur as a complication of an infection with Shiga-toxin (Stx)-producing *Escherichia coli*. Patients typically present with acute kidney injury, microangiopathic hemolytic anemia and thrombocytopenia. There is evidence that Stx-induced renal damage propagates a pro-inflammatory response. To date, therapy is limited to organ-supportive strategies. Bruton’s tyrosine kinase (BTK) plays a pivotal role in recruitment and function of immune cells and its inhibition was recently shown to improve renal function in experimental sepsis and lupus nephritis. We hypothesized that attenuating the evoked immune response by BTK-inhibitors (BTKi) ameliorates outcome in HUS. We investigated the effect of daily oral administration of the BTKi ibrutinib (30 mg/kg) and acalabrutinib (3 mg/kg) in mice with Stx-induced HUS at day 7. After BTKi administration, we observed attenuated disease progression in mice with HUS. These findings were associated with less BTK and downstream phospholipase-C-gamma-2 activation in the spleen and, subsequently, a reduced renal invasion of BTK-positive cells including neutrophils. Only ibrutinib treatment diminished renal invasion of macrophages, improved acute kidney injury and dysfunction (plasma levels of NGAL and urea) and reduced hemolysis (plasma levels of bilirubin and LDH activity). In conclusion, we report here for the first time that BTK inhibition attenuates the course of disease in murine HUS. We suggest that the observed reduction of renal immune cell invasion contributes – at least in part – to this effect. Further translational studies are needed to evaluate BTK as a potential target for HUS therapy to overcome currently limited treatment options.

## 1 Introduction

Food-borne infections with Shiga-toxin (Stx)-producing *Escherichia coli* (STEC) are the major cause of hemolytic-uremic syndrome (HUS). In 2020, 4446 cases of STEC infections were registered in 36 European countries of which 320 patients suffered from HUS (1). Thereby, HUS is a rare, but acute, renal disorder and clinically presents with a triad of acute kidney injury (AKI), microangiopathic hemolytic anemia and thrombocytopenia. With a mortality rate of 3% in young children, HUS survivors can suffer from persisting end-stage renal disease (ESRD) as well as frequently occurring long-term neurological sequelae (2, 3). After translocation of Stx across the human intestinal epithelium, Stx is transferred mainly by immune cells *via* extracellular vesicles to renal endothelial cells, which express the Stx receptor globotriasylceramide (Gb3) (4, 5). Following internalization, Stx exerts ribotoxic effects resulting in an apoptotic and proinflammatory environment. The profound renal injury and tissue destruction caused by Stx results in immune cell migration to kidney, originating from natural reservoirs such as spleen, leading to an amplification of the local and systemic inflammation (5). A specific disease-modifying therapy for HUS is not available and, hence, therapy is currently limited to supportive options including fluid resuscitation and renal replacement therapy (6). Moreover, prospective randomized clinical trials are not feasible due to generally low incidences and unpredictable epidemic HUS outbreaks.

Bruton’s tyrosine kinase (BTK) is a cytoplasmic and non-receptor binding protein tyrosine kinase belonging to the TEC family of kinases. Although the function of the BTK was first described in B-cell development, all cells of hematopoietic origin, excluding T-cells, express BTK indicating its important role in innate and adaptive immunity (7–9). The pivotal role of BTK in the development, recruitment and function of innate immune cells is further highlighted by the fact that BTK-deficient mice or mice treated with BTK inhibitors (BTKi) present with a reduced number of macrophages and migration of immature neutrophil granulocytes to inflamed tissue (10–12).

As BTK takes an intermediary role in diverse signaling pathways *via* downstream targets such as phospholipase-C-gamma-2 (PLCγ2), that influences cell survival, proliferation, differentiation and activation, excessive activation of BTK has been linked to pathophysiological processes in many B-cell-mediated cancers (13). Therefore, intense efforts have been made to design reversible and irreversible BTKi of which the CYS-481-binding inhibitors ibrutinib and second-generation acalabrutinib have been approved by the FDA (14). Interestingly, BTKi also reduce the local and/or systemic inflammation, the associated renal injury and dysfunction in animal models of sepsis, metabolic inflammation and lupus nephritis as well as in patients with COVID-19 (12, 15–17).

The role of BTK in the pathophysiology of STEC-HUS remains unknown. However, the promising results in preclinical studies of inflammatory diseases as well as the findings of the gene expression analysis performed in our well-characterized model of murine HUS (Gene Expression Online - GSE99229) (18) led us to the hypothesis that BTK plays a pivotal role in the inflammatory response and the resulting disease progression in STEC-HUS. Therefore, we investigated the effect of the two FDA-approved, irreversible BTKi ibrutinib and acalabrutinib in a murine model of HUS.

## 2 Material and methods

### 2.1 Animal experiments and study design

The induction of murine HUS by repetitive doses of Stx purified from an O157:H7 EHEC strain 86-24 patient isolate was performed as described previously (18). Male C57BL/6J wild-type mice aged 10-14 weeks were randomly assigned to one of six groups (sham + vehicle; HUS + vehicle; sham + ibrutinib; HUS + ibrutinib; sham + acalabrutinib; HUS + acalabrutinib) (n = 16 per group). Briefly, mice received low doses of Stx (25 ng/kg BW in 0.9% NaCl; HUS groups) or 0.9% NaCl (sham groups) i.v. on day 0, 3 and 6 and BTKi treatment (ibrutinib, acalabrutinib or vehicle) every 24 h p.o. starting immediately after initial Stx injection. For fluid resuscitation, mice received 800 µl Ringer’s Lactate solution subcutaneously three times daily. Body weight was monitored daily and disease progression was evaluated three times daily using an established HUS score (**Supplementary Table S1**), in which the disease severity was categorized from 1 = no signs of illness to 5 = dead. Survival was monitored up to day 7 using humane endpoints (18, 19). Animals were exsanguinated in deep anesthesia (100 mg/kg ketamine; 10 mg/kg xylazine) and perfused with 0.9% NaCl. All *in vivo* experiments were approved by the regional animal welfare committee and the Thuringian State Office for Consumer Protection (registration number UKJ-20-018) and were performed in accordance with German legislation and the approved guidelines.

### 2.2 BTK inhibitors

Ibrutinib and acalabrutinib (MedChemExpress) were diluted to 30 mg/kg BW or 3 mg/kg BW in vehicle, respectively. The vehicle was composed of 5% mannitol (Carl Roth), 0.5% gelantine (Sigma Aldrich) and 2.5% dimethyl sulfoxide (DMSO; Carl Roth) dissolved in injection-grade water (Fresenius Kabi).

### 2.3 Blood and plasma sample analysis

Blood withdrawal and plasma preparation (at 4°C) were performed as described previously (18). Plasma neutrophil gelatinase associated lipocalin (NGAL), urea, bilirubin and plasma activity of lactate dehydrogenase (LDH) were analyzed with commercial kits according to manufacturer’s instructions (**Supplementary Table S2**). Hemograms were determined using scil Vet abc Plus+ (scil animal care company GmbH).

### 2.4 Tissue preparation, histopathology, and immunohistochemical staining

Renal tissue was fixed for at least 72 h in 5% buffered formaldehyde solution (Fischar), before dehydration in descending alcohol solution and embedding in paraffin (Thermo Fisher Scientific) were performed as described previously (18). For all histological staining procedures, 2 µm renal sections were prepared. Histopathological evaluation of renal and splenic tissue using periodic acid Schiff (PAS) staining were performed as described previously (18). Staining for kidney injury molecule-1 (KIM-1), BTK, lymphocyte antigen 6 complex (Ly6g), F4-80, CD3, Ki67 and cleaved caspase-3 (CC-3) were used to evaluate renal sections immunohistochemically. Generally, sections were deparaffinized and hydrated as described previously (18). Blocking of endogenous peroxidase was performed using 3% H_2_O_2_ (Carl Roth) and target retrieval solution (pH 6; Dako) was utilized for antigen retrieval in a pressure cooker. Bovine serum albumin (BSA; Sigma Aldrich) or 20% serum (PAA Laboratories) as well as avidin and biotin solution (15 min each; Vector Laboratories) were used each to block unspecific binding sites (**Supplementary Table S3**). Renal sections were incubated with primary antibody (**Supplementary Table S4**) overnight at 4°C. Sections were further incubated with secondary antibody (**Supplementary Table S5**) and with VectaStain ABC kit (Vector Laboratories) for 30 min each (**Supplementary Table S3** for detailed information). As substrate, 3,3-diaminobenzidine (DAB; Vector Laboratories) was used and sections were counterstained with hemalaun (Carl Roth). Finally, renal sections were dehydrated and mounted for observation. Tris(hydroxymethyl)aminomethan (TRIS) buffer (pH 7.6) containing 50 mM TRIS (Carl Roth), 300 mM sodium chloride (Carl Roth), 0.04% Tween^®^ 20 (Sigma Aldrich) was used to wash renal sections between the staining processes. Staining of thrombocytes (glycoprotein-1b (GP1b)) and fibrin deposition (acid fuchsin–Orange G stain (SFOG)) was performed as described previously (18, 19).

### 2.5 Quantification of histopathology and immunohistochemical staining

PAS staining was used for evaluation of vacuolization of splenic cells. Splenic vacuolization was quantified by counting the number of vacuoles in a grid area (10 x 10 caskets; grid area: 0.0156 mm²) for 20 adjacent areas (magnification 1000x). Quantification and evaluation for staining of KIM-1, F4-80, CD3, Ki67 and CC-3 with a grid area of 0.0977 mm² and 400x magnification was performed as described previously (18). Staining of BTK positive cells was quantified by counting the number of intersections overlapping the positive brown staining in a grid area (10 x 10 caskets; grid area: 0.0977 mm²) for 20 adjacent cortical areas (magnification 400x). Staining of Ly6g was quantified by counting the number of caskets with positive brown staining in a grid area (10 x 10 caskets; grid area: 0.0977 mm²) for 20 adjacent cortical areas (magnification 400x). Images were taken using KEYENCE BZ-X800 microscope and BZ-X800 viewer after performing white balance and auto exposure at magnification of 400x (1000x for spleen). Quantification of thrombocytes and fibrin deposition was performed as described previously (18, 19).

### 2.6 Immunoblot analysis

15 mg of frozen spleen were homogenized in 10 µl/mg lysis buffer as described previously (20). Concentration of proteins was assessed as described previously (20). 15 µg (for pBTK/BTK, NLRP3, pro-IL-1β) and 60 µg (for pPLCγ2/PLCγ2) of protein were reduced in Laemmli buffer at 95°C for 5 min and 300 rpm shaking, loaded onto 10% TGX Stain-Free FastCast gels (Bio-Rad Laboratories) and transferred as described previously (20). Membranes were blocked using 5% BSA in TRIS buffered saline with Tween-20 (TBS-T) at room temperature for 1 h. Membranes were incubated in primary antibody diluted in 5% BSA in TBS-T overnight at 4°C (**Supplementary Table S6**). Membranes were washed 5 times for 5 min each in TBS-T followed by the incubation in HRP-coupled secondary antibodies, diluted in 5% BSA in TBS-T, at room temperature for 1 h (**Supplementary Table S6**). Again, membranes were washed 5 times for 5 min each in TBS-T. Clarity Western ECL substrate (Bio-Rad Laboratories) and the ChemiDoc MP Imaging System (Bio-Rad Laboratories) were used for signal detection. Image Lab software (Bio-Rad Laboratories) was used for analysis of relative protein expression as described previously (20). Bands with normalization factors between 0.7 and 1.3 were considered for evaluation (21). Phosphorylation levels were determined by the division of relative phosphorylated protein by relative pan protein and subsequent normalization to vehicle-treated HUS group.

### 2.7 Statistics

Data were analyzed using GraphPad Prism 7.05 (GraphPad Software) and are depicted as mean + SD for n observations, where n represents the number of animals studied. Survival was analyzed generating Kaplan-Meier curves. Kruskal-Wallis test (non-parametric test) or ordinary one-way ANOVA (parametric test) was used to compare sham groups to their corresponding HUS groups, as well as HUS + vehicle compared with HUS + BTKi. Gaussian distribution was verified using Shapiro-Wilk normality test at 0.05 significance level. If necessary, values were converted to logarithmic values to achieve normality. For immunoblotting, Mann-Whitney-U test was used for comparison between two groups. A P-value < 0.05 was considered significant.

## 3 Results

### 3.1 Disease severity is improved in BTKi-treated mice with HUS

**Figure 1:**
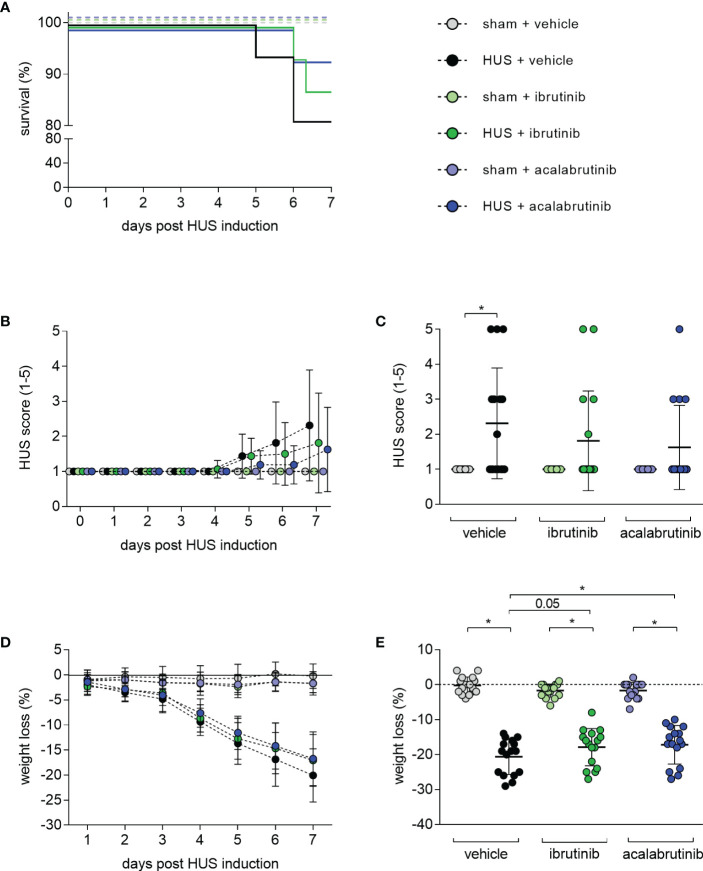
Clinical presentation of mice with HUS treated with ibrutinib or acalabrutinib. HUS followed up for 7 days in sham mice and mice subjected to HUS with daily oral application of vehicle, ibrutinib or acalabrutinib. **(A)** Survival by Kaplan-Meier survival analysis + *post hoc* test, analysis of **(B)** progression of HUS score in the course of the experiment (ranging from 1 = no signs of illness to 5 = dead), **(C)** HUS score on day 7, **(D)** progression of weight loss in the course of the experiment, **(E)** weight loss on humane endpoints or day 7 (n = 16 per group). Data are expressed as **(B, D)** dot blot and **(C, E)** scatter dot blot with mean + SD. **(C)** Kruskal-Wallis test + Dunn’s multiple comparison test, **(E)** ordinary one-way ANOVA + Holm-Sidak’s multiple comparison test. *P < 0.05. HUS, hemolytic uremic syndrome.

Seven-day survival of vehicle-treated mice with HUS (81.25%) was decreased compared with vehicle-treated sham mice (100%) (**Figure 1A**). Mice with HUS treated with ibrutinib (87.5%) or acalabrutinib (93.75%) showed an increased, but not significantly altered, survival rate compared with vehicle-treated mice with HUS. During the experiment, all mice with HUS showed increasing disease progression (i.e. increase in morbidity), indicated by the clinical HUS score (**Figure 1B**). However, the HUS score on day 7 was only significantly increased in vehicle-treated mice with HUS compared with their corresponding sham group (**Figure 1C**). Mice with HUS of all groups lost a significant amount of weight during the 7-day experimental period (**Figure 1D**) compared with their corresponding sham group. Weight loss was highest in vehicle-treated mice with HUS (20.6%), followed by mice with HUS treated with acalabrutinib (17.2%) (**Figure 1E**). Comparison of the HUS groups revealed that mice with HUS treated with acalabrutinib lost significantly less weight compared with the vehicle-treated mice with HUS, while weight loss of ibrutinib-treated mice with HUS was reduced (P = 0.05), but not significantly altered, compared with the vehicle-treated HUS group.

### 3.2 Kidney injury, proliferation and apoptosis are partly ameliorated by BTKi treatment in mice with HUS

**Figure 2:**
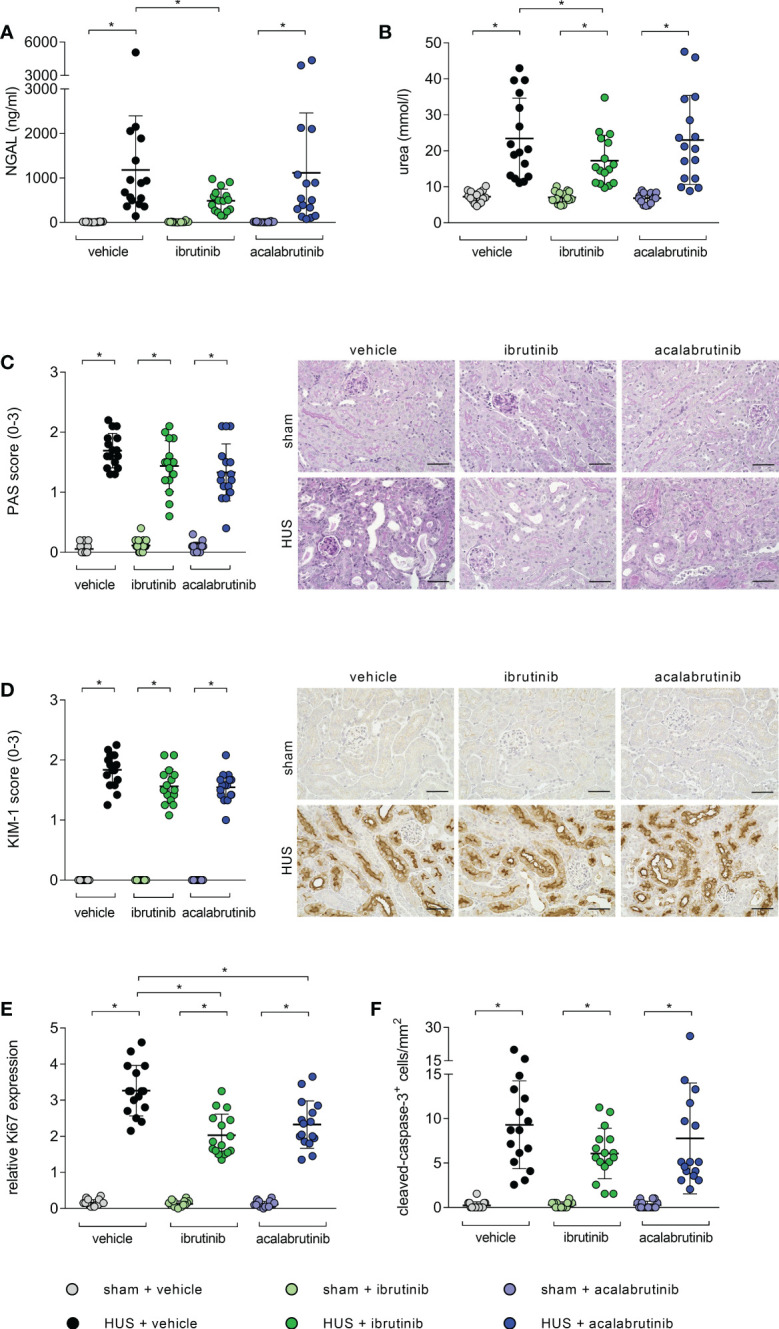
Parameters of kidney injury, proliferation and apoptosis of mice with HUS treated with ibrutinib or acalabrutinib. Determination of plasma **(A)** NGAL and **(B)** urea (n = 16 per group) on humane endpoint or day 7. Quantification and representative images of **(C)** PAS reaction and **(D)** relative KIM-1 expression in renal sections (n = 16 per group) on humane endpoint or day 7. Bars = 50 µm (400x magnification). Quantification of renal **(E)** Ki67 and **(F)** CC-3 expression (n = 16 per group) on humane endpoint or day 7. Data are expressed as scatter dot plot with mean + SD. **(A, B, E)** ordinary one-way ANOVA + Holm-Sidak’s multiple comparison test, **(C, D, F)** Kruskal-Wallis test + Dunn’s multiple comparison test. *P < 0.05. NGAL, neutrophil gelatinase-associated lipocalin; PAS, periodic acid Schiff; KIM-1, kidney injury molecule-1; HUS, hemolytic-uremic syndrome.

Mice with HUS treated with vehicle or acalabrutinib showed severe renal injury, indicated by significantly increased plasma NGAL compared with their corresponding sham group (**Figure 2A**). In contrast, ibrutinib-treated mice with HUS showed elevated, but not significantly altered, plasma NGAL compared with their corresponding sham group as well as significantly less plasma NGAL compared with vehicle-treated mice with HUS. Mice with HUS of all groups showed significantly increased plasma urea compared with their corresponding sham group (**Figure 2B**). However, ibrutinib-treated mice with HUS showed significantly less plasma urea when compared with mice with HUS of the vehicle group. For further evaluation of tissue damage, PAS staining and analysis of KIM-1 expression was performed. Mice with HUS of all groups showed a significantly higher PAS score (**Figure 2C**) and KIM-1 (**Figure 2D**) score compared with their corresponding sham group. However, the highest PAS and KIM-1 scores were measured in vehicle-treated mice with HUS, while ibrutinib- and acalabrutinib-treated mice with HUS tended to have lower PAS and KIM-1 scores. Proliferation and apoptosis in renal tissue was evaluated using Ki67 and CC-3 staining, respectively. Mice with HUS of all groups showed significantly increased cell proliferation compared with their corresponding sham group (**Figure 2E**; **Supplementary Figure 1A**). Notably, treatment with ibrutinib or acalabrutinib significantly reduced the amount of proliferated cells in renal tissue compared with vehicle-treated mice with HUS. Similarly, mice with HUS of all groups showed significantly increased number of apoptotic cells compared with their corresponding sham group irrespective of their treatment (**Figure 2F**; **Supplementary Figure 1B**).

### 3.3 Surrogate parameters of hemolysis and fibrin deposition are reduced in ibrutinib-treated mice with HUS

**Figure 3:**
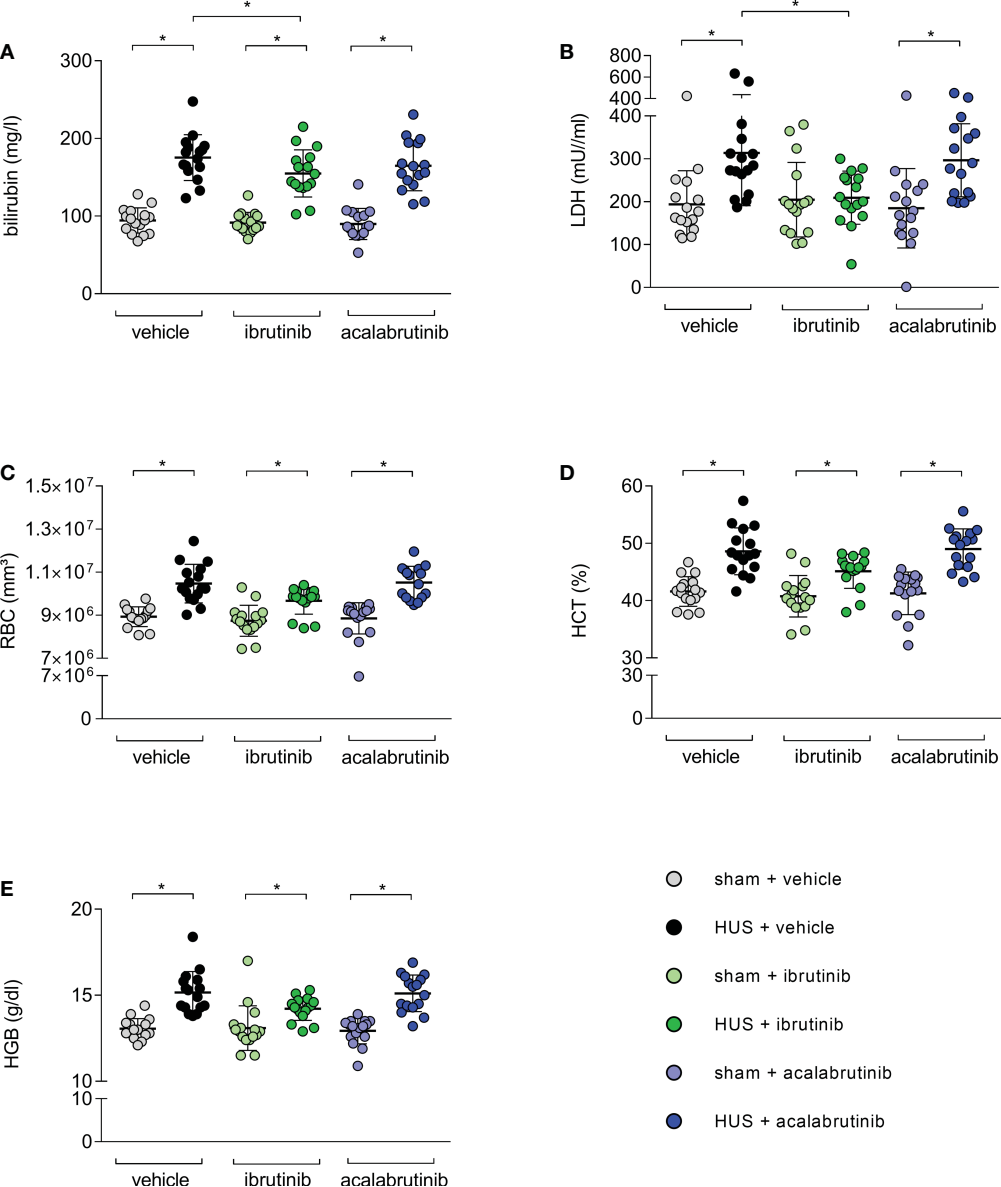
Surrogate parameters of hemolysis of mice with HUS treated with ibrutinib or acalabrutinib. Determination of plasma **(A)** bilirubin; **(B)** LDH activity and whole blood **(C)** erythrocytes, **(D)** hematocrit and **(E)** hemoglobin (n = 16 per group) on humane endpoint or day 7. Data are expressed as scatter dot plot with mean + SD. **(A)** ordinary one-way ANOVA + Holm-Sidak’s multiple comparison test, **(B–E)** Kruskal-Wallis test + Dunn’s multiple comparison test. *P < 0.05. RBC, erythrocytes; HCT, hematocrit; HGB, hemoglobin; LDH, lactate dehydrogenase; HUS, hemolytic-uremic syndrome.

As indirect hemolysis markers, plasma bilirubin and plasma LDH activity were measured. Mice with HUS of all groups showed significantly higher bilirubin levels compared with their corresponding sham group (**Figure 3A**). However, ibrutinib-treated mice with HUS showed significantly reduced plasma bilirubin when compared with the mice with HUS of the vehicle group. Similar results were seen for measurement of LDH activity in plasma. Mice with HUS treated with vehicle or acalabrutinib showed increased plasma LDH activity compared with their corresponding sham group (**Figure 3B**), while ibrutinib-treated mice with HUS showed LDH activity equal to those seen in the corresponding sham animals. All mice with HUS showed significantly increased erythrocyte count (**Figure 3C**), hematocrit (**Figure 3D**) and hemoglobin (**Figure 3E**) in whole blood compared with their corresponding sham group, indicating the development of hemoconcentration as described previously (18). An insignificant trend towards lower mean values of hemoconcentration indicators was observed in ibrutinib-treated mice with HUS compared with vehicle-treated mice with HUS. Significant thrombocytopenia was not observed in mice with experimental HUS regardless of treatment (**Supplementary Figure S2A**). Surrogate parameters of red blood indices (mean corpuscular volume, mean corpuscular hemoglobin, mean corpuscular hemoglobin concentration) did not show any differences between vehicle-treated groups and BTKi-treated groups, indicating that treatment with BTKi in mice did not influence characteristics of erythrocytes in mice (**Supplementary Figures S2B–D**). To analyze thrombotic microangiopathy in the kidneys, thrombocytes counts in renal sections of all groups were assessed by GP1b staining, and fibrin deposition was assessed by SFOG staining. Renal thrombocyte counts were significantly increased in the kidney sections of all mice with HUS compared with the corresponding sham mice, regardless of treatment (**Supplementary Figure S3A**). Fibrin deposits were significantly increased in the kidney sections of vehicle- or acalabrutinib-treated mice with HUS compared with the corresponding sham mice, however not in ibrutinib-treated mice (**Supplementary Figure S3B**).

### 3.4 Dampened renal immune cell invasion by treatment with BTKi in mice with HUS

**Figure 4:**
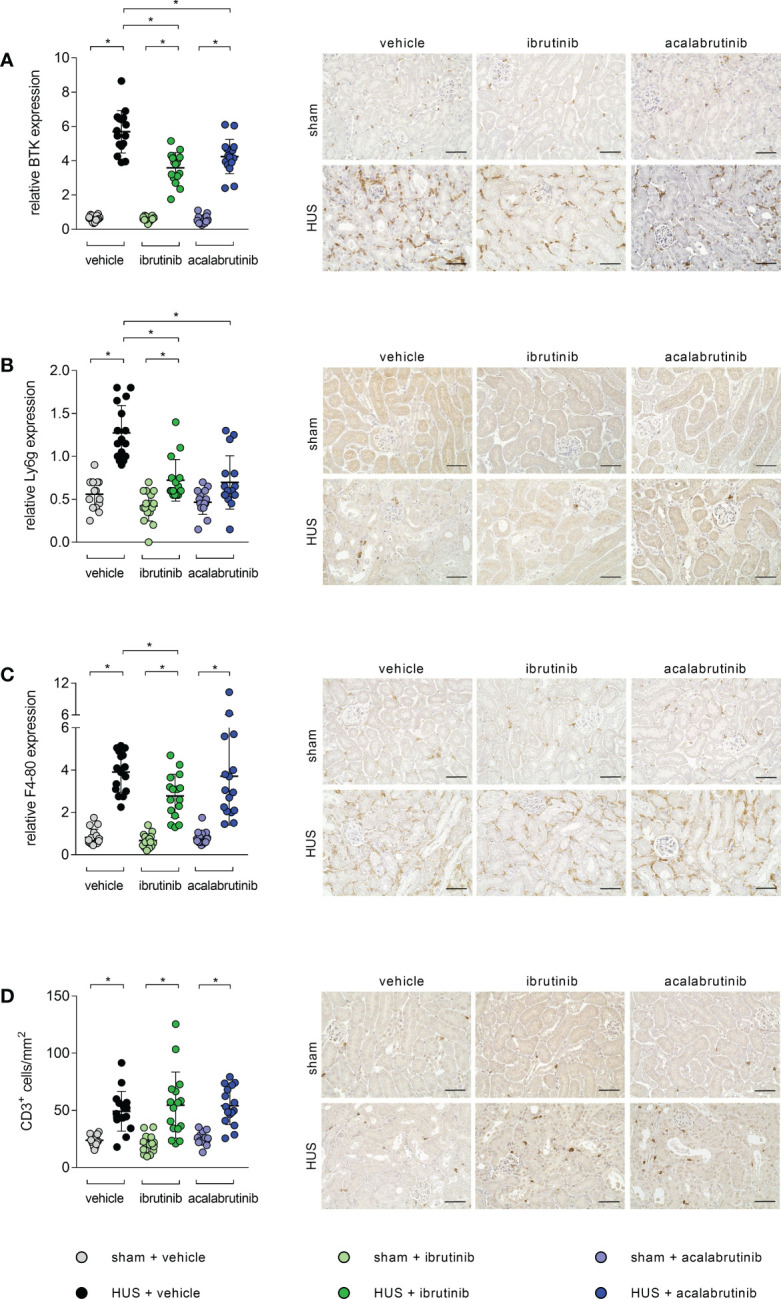
Renal immune response of mice with HUS treated with ibrutinib or acalabrutinib. Quantification and representative images of **(A)** BTK, **(B)** Ly6g, **(C)** F4-80 and **(D)** CD3 expression in renal sections (n = 16 per group) on humane endpoint or day 7. Bars = 50 µm (400x magnification). Data are expressed as scatter dot plot with mean + SD. **(A, C, D)** ordinary one-way ANOVA + Holm-Sidak’s multiple comparison test. **(B)** Kruskal-Wallis test + Dunn’s multiple comparison test. *P < 0.05. BTK, Bruton’s tyrosine kinase; Ly6g, lymphocyte antigen 6 complex, locus G; HUS, hemolytic-uremic syndrome.

BTK is mainly expressed in infiltrating immune cells. Therefore, histological analysis for renal immune cell invasion was performed. In all stainings, mice with HUS of all groups showed significant increase of the evaluated immune cells compared with their corresponding sham group. However, histological staining for BTK revealed a significant reduction of BTK-positive cells in ibrutinib- and acalabrutinib-treated mice with HUS compared with mice with HUS of the vehicle group (**Figure 4A**). Moreover, the same effect was detected for the invasion of neutrophil granulocytes, indicated by Ly6g staining (**Figure 4B**). Quantification of F4-80 (surface marker for macrophages) revealed significantly reduced accumulation of macrophages only in ibrutinib-treated mice with HUS compared with vehicle-treated mice with HUS, while this effect was not seen for acalabrutinib treatment (**Figure 4C**). By contrast, the invasion of CD3-positive cells (surface marker for T-lymphocytes) remained unaffectedly high in mice with HUS of all groups irrespective of their treatment (**Figure 4D**).

### 3.5 Alternated BTK-downstream signaling and grade of vacuolization by BTKi treatment in mice with HUS

**Figure 5:**
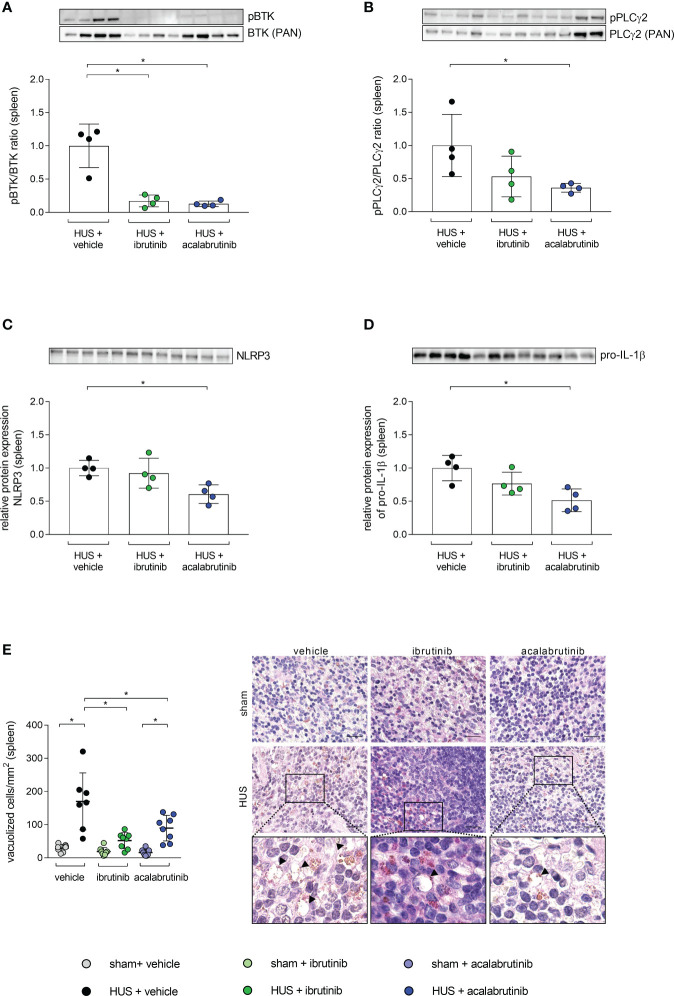
BTK-downstream signaling as well as vacuolization of cells in spleen of mice with HUS treated with ibrutinib or acalabrutinib. Ratio of relative phosphorylated protein expression to relative pan protein expression of **(A)** BTK and **(B)** PLCγ2 as well as relative protein expression of **(C)** NLRP3 and **(D)** pro-IL-1β in spleen (n = 4 per HUS group) in whole splenic protein lysates on humane endpoint or day 7. Quantification and representative images of **(E)** vacuolization of cells in splenic sections (n = 7 - 8 per group) on humane endpoint or day 7. Bars = 20 µm (1000x magnification). Arrows indicate sites of vacuolization. Data are expressed as scatter dot plot with mean + SD. **(A–D)** Mann-Whitney-U test. **(E)** ordinary one-way ANOVA + Holm-Sidak’s multiple comparison test. *P < 0.05. BTK, Bruton’s tyrosine kinase; PLCγ2, phospholipase-C-gamma2; HUS, hemolytic-uremic syndrome; NLRP3, NLR family pyrin domain containing 3; IL-1β, Interleukin-1beta.

As the spleen is described as a natural reservoir of immune cells and BTK is known to have a major impact in the activation of immune cells (9, 22–24), the phosphorylation level of BTK was analyzed in whole-cell protein lysates. BTK phosphorylation was significantly reduced in mice with HUS treated with ibrutinib or acalabrutinib compared with vehicle-treated mice with HUS (**Figure 5A**). As a direct downstream target of BTK, phosphorylation level of PLCγ2 was analyzed in spleen. Ibrutinib-treated mice with HUS showed reduced, but not significantly altered, PLCγ2 phosphorylation compared with vehicle-treated mice with HUS (**Figure 5B**). In contrast, only acalabrutinib treatment in mice with HUS led to significantly reduced phosphorylation of PLCγ2 compared with the vehicle-treated mice with HUS. Since BTK has been described as an activator of the NLRP3 inflammasome, the relative protein expression of NLRP3 and pro-IL-1β in spleen tissue was analyzed. In the spleen, ibrutinib-treated mice with HUS showed a trend towards decreased NLRP3 (**Figure 5C**) and pro-IL-1β (**Figure 5D**) expression, while acalabrutinib-treated mice with HUS showed a significantly decreased protein expression of NLRP3 (**Figure 5C**) and pro-IL-1β (**Figure 5D**) compared with vehicle-treated mice with HUS. For analysis of spleen morphology, PAS staining was performed and vacuolization of cells was counted. Vehicle- and acalabrutinib-treated mice with HUS showed significantly increased vacuolization of cells compared with their corresponding sham group (**Figure 5E**). Moreover, treatment with ibrutinib and acalabrutinib lead to significantly reduced vacuolization of cells in mice with HUS compared with vehicle-treated mice with HUS.

## 4 Discussion

STEC-HUS is a rare, but life-threatening, disease causing acute kidney injury often followed by long-term sequelae. Hitherto, supportive therapy during acute phase of this disease is the mainstay and targeted therapy is still missing. In the present study, we report for the first time that the two FDA-approved BTKi ibrutinib and acalabrutinib attenuate disease progression seen by a reduction of renal immune cell infiltration in a murine model of HUS.

Critical steps in HUS pathophysiology, caused by Stx, are acute kidney injury, microangiopathic hemolytic anemia and thrombocytopenia as well as the evoked immune response. As the kidneys are the main target of Stx in HUS, we investigated the impact of the BTKi ibrutinib and acalabrutinib on renal pathology in our murine HUS model. Ibrutinib-treatment in mice with HUS slightly alleviated renal injury by reducing plasma NGAL and urea as well as proliferation of cells in renal tissue. Nevertheless, a profound improvement in renal tissue damage and dysfunction has not been observed for both inhibitors in the present study. Conversely, ibrutinib and acalabrutinib have already been proven to ameliorate renal dysfunction in murine models of cecal ligation and puncture (CLP)-induced sepsis, sepsis-induced acute kidney injury as well as lupus nephritis (15, 16, 25). In the light of these studies, we hypothesize that inhibition of BTK in the pathophysiology of HUS does not primarily affect the ribotoxic function and subsequent destruction of renal tissue by Stx. Therefore, we analyzed the influence of BTKi on the aspect of hemolysis in the pathophysiology of HUS. In our HUS model, mice suffer from profound dehydration, indicated by increased hemoglobin and hematocrit, and show elevated hemolysis markers, which have been described before (18). In the present study, ibrutinib-treated mice with HUS showed positive impact on hemolysis while intervention with acalabrutinib did not. Interestingly, both inhibitors have already been described as therapeutic option for autoimmune hemolytic anemia and are currently tested in clinical stage 2 trials (ibrutinib: NCT03827603, NCT04398459; acalabrutinib: NCT-04657094) (26, 27). These contradicting results might be explained by different application regimes of ibrutinib (30 mg/kg BW) and acalabrutinib (3 mg/kg BW) in our study. Moreover, potential off-target activity of ibrutinib cannot be excluded. Ibrutinib has been shown to block other TEC family kinases such as epidermal growth factor receptor (EGFR), targeting interleukin-2 inducible T-cell kinase (ITK) and TEC. In contrast, acalabrutinib possesses a much higher selectivity, but reduced potency, for BTK compared with ibrutinib (27–29). Thrombocytopenia is another hallmark of HUS and we already previously described that mice with HUS did not develop a pronounced thrombocytopenia in the employed model. We hypothesized earlier that thrombocytopenia might be masked by hypovolemia and consecutive hemoconcentration (18). Recently published studies have reported antithrombotic properties of low doses of BTKi in mouse models and human volunteers (30–32). Therefore, we investigated the effects of BTKi on thrombotic microangiopathy in HUS by staining platelets and fibrin deposition. In our model acalabrutinib had no effect on surrogate parameters of thrombotic events. However, in ibrutinib-treated mice with HUS, we observed a trend towards less fibrin deposition. Taken together, we observed an advantage for ibrutinib over acalabrutinib in the analysis of renal injury, hemolysis and fibrin deposition in our murine HUS model. However, the improvement of disease progression of HUS by BTKi, seen by decreased morbidity and lowered mortality, was observed for both inhibitors. As BTK is mainly expressed in cells of hematopoietic origin, we assume that treatment with ibrutinib and acalabrutinib mostly affects the evoked immune response after initial renal injury.

In the last decade, intense research has been performed to demonstrate that inflammation is a central part in the pathophysiology of HUS and not just an epiphenomenon (33). Renal destruction, induced by Stx, not only causes vascular damage but also the induction of cytokine and chemokine expression. Thereby, the secretion of interleukin-8 or monocyte chemotactic protein 1 of the Stx-affected cells generates an inflammatory environment, while simultaneously enhancing immune cell adhesion and their migration into renal tissue (33–35). Therefore, it is not surprising that recruitment of macrophages, neutrophils and other immune cells is well documented in murine kidneys of our HUS model as well as in HUS patients (18, 19, 36). Interestingly, binding of Stx to neutrophils prolongs their lifespan, increases reactive oxygen species production and neutrophil extracellular trap formation and thereby intensifying the inflammatory process, indicating neutrophils are an important player in HUS pathophysiology (37). In the present study, we observed that treatment with the BTKi ibrutinib and acalabrutinib significantly reduced the general amount of BTK-positive cells in renal tissue. Further analysis showed that especially neutrophils and macrophages (ibrutinib only) were affected by BTKi treatment in HUS, matching the fact that expression of BTK in both cell types was proven to play an important part in their maturation and function. Matching this, BTK deficiency has been associated with reduced number of monocytes, macrophages as well as granulocytes arresting in a pre-mature stage causing neutropenia in different studies (9, 11, 38, 39). We hypothesize that especially the reduced amount of neutrophils migrating into renal tissue through BTKi is the reason for the improved morbidity and mortality observed in the present study. Underlining this, Lill et al. recently observed that in mice with HUS, tissue-resident renal macrophages produce TNF-α, which is a crucial chemotactic molecule for recruitment of neutrophils (40). TNF-α depletion of these macrophages significantly reduced neutrophil migration into the kidney and significantly improved disease outcome (40). In addition, the functional state of neutrophils has been correlated with renal dysfunction and disease outcome in children with HUS, emphasizing a crucial role of neutrophils in the progression of this disease (36). Besides the role of BTK in the physiological function of immune cells, BTK has also a profound impact on the recruitment process of neutrophils and macrophages from the bloodstream or natural reservoirs into injured tissue, as BTK has been found to play a key role in E-selectin-mediated slow rolling and transmigration of neutrophils into tissue (41, 42). The concept of the spleen as a natural reservoir of immune cells is well established. Neutrophils can contribute to the marginating pool of granulocytes through localization in the spleen in absence of infection or injury (23). Especially the mobilization of splenic monocytes to injured tissue after coronary ligation highlights the ability of the spleen to make immune cells available after tissue destruction (24). To examine, whether inhibition of BTK affects other organs involved in the triggered immune cell activation and recruitment, we analyzed the activation status of splenic BTK and its direct downstream target PLCγ2 (43, 44) and observed a reduced activation of both proteins in splenic tissue by BTKi in mice with HUS. Furthermore, BTK has previously been described as a positive and direct regulator of the NLRP3 inflammasome and the production of pro-inflammatory IL-1β (45–48). We analyzed the protein expression of NLRP3 and pro-IL-1β and observed reduced expression of these proteins in spleen tissue of BTKi-treated mice with HUS. Therefore, we hypothesize that the BTK-downstream signaling, which leads to activation and recruitment of immune cells to the kidney and generation of pro-inflammatory stimuli, is blocked by the employed BTKi, particularly in the spleen of mice with HUS. Moreover, we observed vacuolization of cells in the red pulp of the spleen after HUS induction, which is significantly reduced by BTKi. To our knowledge, this phenomenon has not been described before in the pathophysiology of HUS. Extensive vacuolization in the red pulp has already been negatively associated with the outcome in studies analyzing murine infections with *Plasmodium berghei* or toxicity testing of elmiron (49, 50). Concluding this, we hypothesize that an improved outcome of HUS progression through BTKi could be achieved by a reduced immune cell recruitment from spleen to kidney and a reduced alternation of splenic tissue.

Hitherto, HUS therapy is limited to organ-supportive strategies. We report here for the first time that the commercially available BTKi ibrutinib and acalabrutinib alleviate disease progression in murine HUS by reducing renal immune cell invasion. Moreover, our results indicate less BTK-induced immune cell activation in spleen as potential explanation. The promising results of BTK as a new target in HUS therapy in our study are worth to be further validated.

## Data availability statement

The original contributions presented in the study are included in the article/**Supplementary Material**. Further inquiries can be directed to the corresponding author.

## Ethics statement

The animal study was reviewed and approved by the regional animal welfare committee and the Thuringian State Office for Consumer Protection (registration number UKJ-20-018).

## Author contributions

SMC designed, planned, and supervised the study. SK and SMC wrote the manuscript. SK, BW, SD, and NK performed animal experiments. SK performed data analysis of animal experiments. SK performed and analyzed ELISA, histology, immunohistochemistry, and Western blots. CD performed staining of SFOG, staining and analysis of GP1b. FG provided Shiga toxin. SMC, SK, BW, SD, NK, CT, CD, KA, and FG provided important intellectual content and revised the manuscript. All authors contributed to the article and approved the submitted version.
