# Supplementary material for: Bruton’s tyrosine kinase inhibition attenuates disease progression by reducing renal immune cell invasion in mice with hemolytic-uremic syndrome
Source: Front Immunol. 2023 Feb 23;14:1105181. doi: 10.3389/fimmu.2023.1105181 (PMC9995712; doi:10.3389/fimmu.2023.1105181)
Supplement: Supplementary file 1 [file DataSheet_1.pdf]

Supplementary Material

Bruton's tyrosine kinase inhibition attenuates disease progression by reducing renal immune cell invasion in mice with hemolytic-uremic syndrome

Sarah Kröller, M.Sc.[1,2], Bianka Wissuwa, Ph.D.[1,2], Sophie Dennhardt, M.Sc.[1,2], Nadine Krieg, Ph.D.[1,2], Prof. Christoph Thiemermann, M.D., Ph.D.[3], Prof. Christoph Daniel, M.D., Ph.D.[4], Prof. Kerstin Amann, M.D.[4], Prof. Florian Gunzer M.D.[5], Prof. Sina M. Coldewey, M.D., Ph.D.[1,2,6]

[1]Department of Anesthesiology and Intensive Care Medicine, Jena University Hospital, Jena, Germany

[2]Septomics Research Center, Jena University Hospital, Jena, Germany

[3]William Harvey Research Institute, Barts and The London School of Medicine and Dentistry, Queen Mary University of London, London, United Kingdom

[4]Department of Nephropathology, Friedrich-Alexander University (FAU) Erlangen-Nürnberg, Erlangen, Germany

[5]Department of Hospital Infection Control, University Hospital Carl Gustav Carus, TU Dresden; Dresden, Germany

[6]Center for Sepsis Control and Care (CSCC), Jena University Hospital, Jena, Germany

*** Correspondence:**
Sina M. Coldewey

sina.coldewey@med.uni-jena.de

Keywords: hemolytic-uremic syndrome, acute kidney injury, inflammation, animal model, Bruton's tyrosine kinase, ibrutinib, acalabrutinib

1 Supplementary Tables

Table S1: HUS score

Each criterion was monitored three times daily. Additionally, after two times consecutive score 3, the mice were checked one more time. Grade of disease was calculated from the sum of all points of all criteria.

score	points/ criteria	I. activity	II. reaction	III. posture	IV. general symp- toms	V. neuro- logical symp- toms	VI. fur
**score 1** **no signs of illness** **0 points**	0	active, strong	curious, fast movement s	normal	none	none	shiny, even
**score 2** **low-grade** **1 – 4 points**	1	lower activity, occasional interruptio ns	reduced attention, appropriate reaction				blunt, adjacent
**score 3** **mid-grade** **5 – 12 points**	2	markedly reduced	reduced attention, delayed reaction	slightly hunched	loss of 10-20% BW within 48 h	beginning hind limb clasping	blunt, slightly ruffled
**score 4** **high-grade** **≥ 12** **points**	3	lethargic, no movement s	none	strong hunch	loss of > 20% BW within 48 h	pronounce d hind limb clasping	blunt, strong pilo-erection
**score 5** **dead**	n/a	n/a	n/a	n/a	n/a	n/a	n/a

*BW: body weight, n/a: not available

Table S2: Commercial kits

target	name	supplier	cat. No.	batch
**plasma NGAL**	LEGEND MAX™ Mouse NGAL (Lipocalin-2) ELISA Kit	BioLegend, Inc.	443707	B320222
**plasma urea**	Urea Assay Kit	Abcam plc.	ab83362	GR3390168-1
**plasma bilirubin**	Bilirubin (Total and Direct) Colorimetric Assay Kit	BioVision, Inc.	K553-100	6A10K05530
**plasma LDH**	LDH Assay Kit / Lactate Dehydrogenase Assay Kit (Colorimetric)	Abcam plc.	ab102526	GR3274914-1

Table S3: Information for immunohistochemistry protocol details

Blocking time of endogenous peroxidase, time of antigen retrieval, details of blocking as well as working dilution and diluent for 1st and 2nd antibodies are indicated.

staining	blocking of endogenous peroxidase (3% H_2O_2)	antigen retrieval (pH=6; 110 °C)	blocking	1st AB	2nd AB
**KIM-1**	20 min	10 min	1% BSA in TB; 1 h	1:1000 in 1% BSA in TB	1:500 in TB
**BTK**	20 min	15 min	20% NGS in 1% BSA in TB; 1 h	1:200 in 1% BSA in TB	1:200 in TB
**Ly6g**	15 min	10 min	20% NRS in 1% BSA in TB; 30 min	1:100 in 1% BSA in TB	1:200 in 1% BSA in TB
**F4-80**	20 min	10 min	20% NRS in 1% BSA in TB; 30 min	1:100 in 1% BSA in TB	1:200 in TB + 2% NMS
**CD3**	10 min	10 min	20% NGS in 1% BSA in TB; 30 min	1:100 in TB	1:200 in TB + 2% NMS
**Ki67**	20 min	10 min	20% NGS in 1% BSA in TB; 1 h	1:200 in TB	1:200 in TB
**CC-3**	20 min	5 min	1% BSA in TB; 1 h	1:200 in TB	1:200 in TB
**GP1b**	n/a	n/a	20% NRS in 5% skim milk; 1 h	1:1000 in TB	n/a

*BSA (bovine serum albumin); NGS (normal goat serum); NMS (normal mouse serum); NRS (normal rabbit serum); TB (TRIS buffer)

Table S4: Primary antibodies used for immunohistochemistry

Supplier, batch and catalogue number are indicated.

antibody	supplier	cat. No.	batch
**polyclonal goat anti-KIM-1**	R&D Systems, Inc.	AF1817	KCA0318012
**monoclonal rabbit anti-BTK**	Cell Signaling Technology, Inc.	8547	13
**monoclonal rat anti-Ly6g**	Abcam plc.	Ab25377	GR3280757-2
**monoclonal rat anti-F4-80**	Bio-Rad Laboratories, Inc.	MCA497	1702
**monoclonal rabbit anti-CD3**	Zytomed GmbH	RBK024-05	W337/ A2135
**monoclonal rabbit anti-Ki67**	Thermo Fischer Scientific Inc.	RM-9106-S	9106S 1607Z1
**polyclonal rabbit anti-caspase-3 (cleaved)**	Zytomed GmbH	RBK009-05	X048
**monoclonal rat anti-GP1b**	Emfret Analystics GmbH	M040-0	n/a

Table S5: Secondary antibodies used for immunohistochemistry

Supplier, batch and catalogue number are indicated.

antibody	supplier	cat. No.	batch
**biotinylated anti-rabbit IgG (H+L)**	Vector Laboratories, Inc.	BA-1000	ZE0730
**biotinylated anti-goat IgG (H+L)**	Vector Laboratories, Inc.	BA-5000	ZA0425
**biotinylated anti-rat IgG (H+L)**	Vector Laboratories, Inc.	BA-4001	ZE0201

Table S6: Primary and secondary antibodies used for immunoblotting

Supplier, batch and catalogue number are indicated.

antibody	supplier	cat. No.	batch	working dilution
**monoclonal rabbit anti-BTK**	Cell Signaling Technology, Inc.	8547	13	1:1000 in 5% BSA + TBS-T
**monoclonal rabbit anti-phospho-BTK**	Cell Signaling Technology, Inc.	87141	1	1:1000 in 5% BSA + TBS-T
**monoclonal mouse anti-IL-1β**	Cell Signaling Technology, Inc.	12242	4	1:2000 in 5% BSA + TBS-T
**monoclonal mouse anti-NLRP3**	AdipoGen Life Sciences	AG-20B-0014	AK1812012	1:1000 in 5% BSA + TBS-T
**polyclonal rabbit anti-PLC-gamma2**	Novus Biologicals	NBP1-87558	A106892	1:1000 in 5% BSA + TBS-T
**polyclonal rabbit anti-phospho-PLC-gamma2**	Cell Signaling Technology, Inc.	3871	5	1:1000 in 5% BSA + TBS-T
**anti-mouse IgG HRP-linked**	Cell Signaling Technology, Inc.	7076	38	1:2000 in 5% BSA + TBS-T
**anti-rabbit IgG, HRP-linked**	Cell Signaling Technology, Inc.	7074S	30	1:3000 in 5% BSA + TBS-T

*BSA (bovine serum albumin); TBS-T (Tris-buffered saline with Tween20)

2 Supplementary Figures

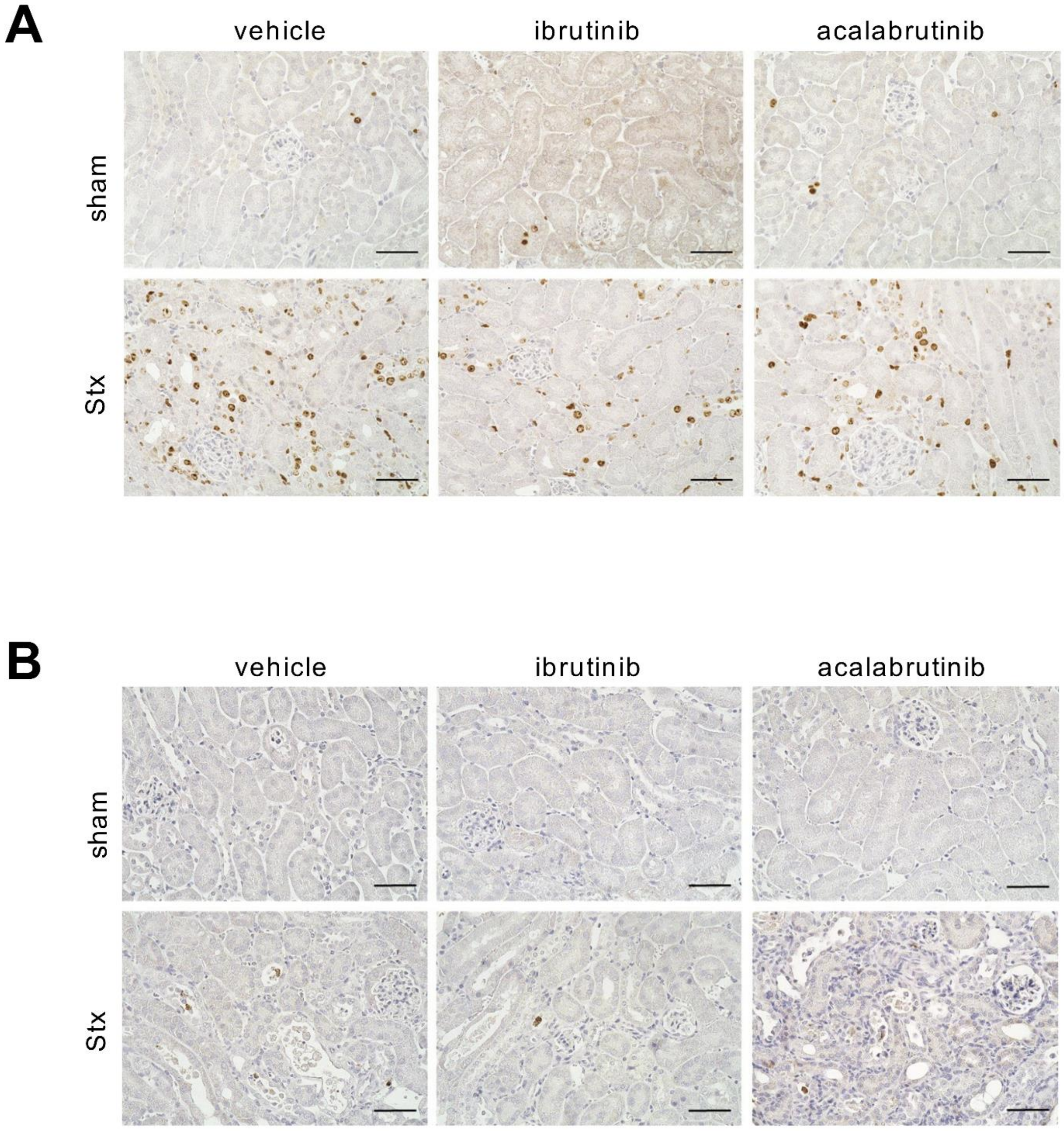

Figure S1: Ki67 and CC-3 staining of mice with experimental HUS treated with ibrutinib or acalabrutinib. Representative images of renal (A) Ki67 and (B) CC-3 staining of sham mice and mice subjected to HUS (n = 16 per group) on humane endpoint/ day 7. Bars = 50 µm (magnification 400x). Quantifications are shown in Figures 2E (Ki67) and 2F (CC-3). CC-3: cleaved-caspase 3; HUS: hemolytic-uremic syndrome

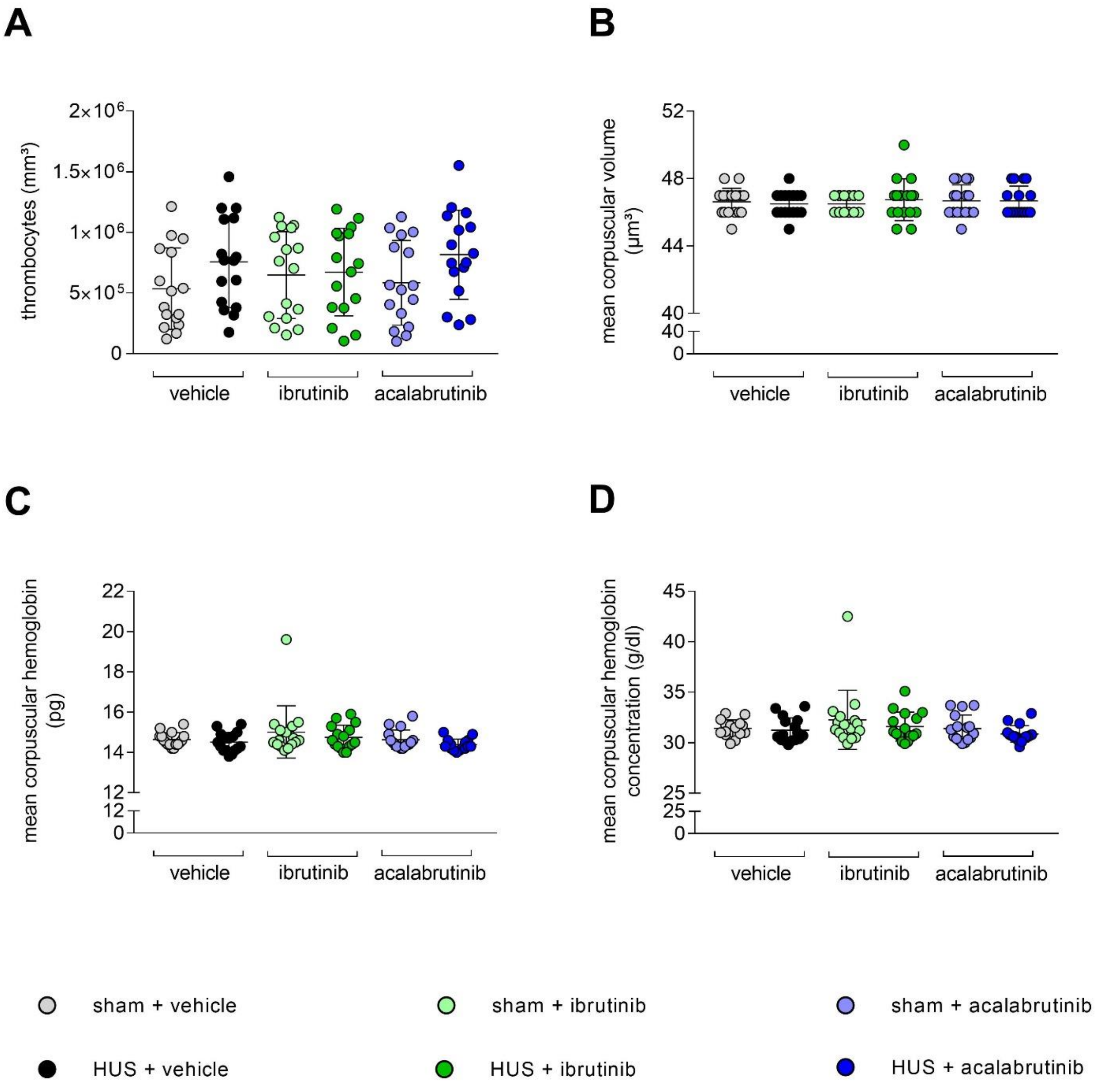

Figure S2: Surrogate parameters of thrombocytes and erythrocytes of mice with HUS treated with ibrutinib or acalabrutinib. Determination of whole blood (A) thrombocytes, (B) mean corpuscular volume, (C) mean corpuscular hemoglobin, (D) mean corpuscular hemoglobin concentration (n = 16 per group) on humane endpoint or on day 7. Data are expressed as scatter dot plot with mean $\pm$ SD. (A-C) Kruskal-Wallis test + Dunn's multiple comparison test. HUS: hemolytic-uremic syndrome

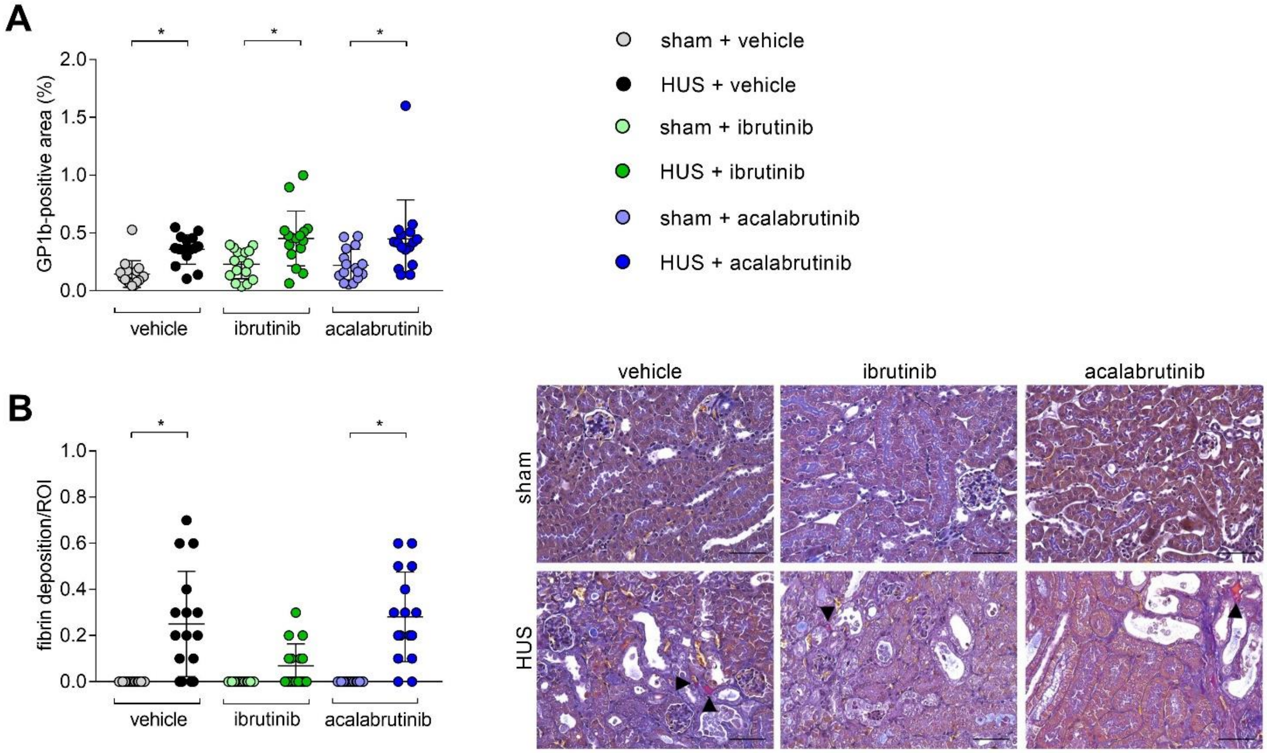

Figure S3: Markers of thrombotic microangiopathy in kidney of mice with HUS treated with ibrutinib or acalabrutinib. Quantification of (A) thrombocytes in renal sections (n = 15-16 per group) on humane endpoint or day 7. Quantification and representative images of (B) fibrin depositions in renal sections (n = 16 per group) on humane endpoint or day 7. Bars = 50 µm (400x magnification). Arrows indicate sites of fibrin deposition. Data are expressed as scatter dot plot with mean + SD. (A+B) Kruskal-Wallis test + Dunn's multiple comparison test. *P < 0.05. HUS: hemolytic-uremic syndrome; GP1b: glycoprotein 1b
